# Characterization of soil nematode communities in three cropping systems through morphological and DNA metabarcoding approaches

**DOI:** 10.1038/s41598-018-20366-5

**Published:** 2018-01-31

**Authors:** Amy M. Treonis, Samantha K. Unangst, Ryan M. Kepler, Jeffrey S. Buyer, Michel A. Cavigelli, Steven B. Mirsky, Jude E. Maul

**Affiliations:** 1 Department of Biology, University of Richmond, Richmond, VA 23173 USA; 2 USDA-ARS, Sustainable Agricultural Systems Laboratory, Beltsville, MD 20705 USA

## Abstract

We used complementary morphological and DNA metabarcoding approaches to characterize soil nematode communities in three cropping systems, conventional till (CT), no-till (NT) and organic (ORG), from a long-term field experiment. We hypothesized that organic inputs to the ORG system would promote a more abundant nematode community, and that the NT system would show a more structured trophic system (higher Bongers MI) than CT due to decreased soil disturbance. The abundance of Tylenchidae and Cephalobidae both showed positive correlations to soil organic carbon and nitrogen, which were highest in the ORG system. The density of omnivore-predator and bacterial-feeding nematodes was reduced in NT soils compared to CT, while some plant-parasitic taxa increased. NT soils had similar Bongers MI values to CT, suggesting they contained nematode communities associated with soils experiencing comparable levels of disturbance. Metabarcoding revealed within-family differences in nematode diversity. Shannon and Simpson’s index values for the Tylenchidae and Rhabditidae were higher in the ORG system than CT. Compared to morphological analysis, metabarcoding over- or underestimated the prevalence of several nematode families and detected some families not observed based on morphology. Discrepancies between the techniques require further investigation to establish the accuracy of metabarcoding for characterization of soil nematode communities.

## Introduction

Soil ecosystems harbor diverse assemblages of prokaryotes and eukaryotes that play vital roles in ecosystem functions, including decomposition and nutrient cycling^1,2^. Soil biota have shown sensitivity to agricultural management strategies (e.g., tillage, herbicide and pesticide application, organic amendments)^3–7^. Furthermore, compared to natural ecosystems, arable soils generally undergo a reduction in biodiversity and ecosystem function that is magnified by agricultural intensification, but these changes are not fully understood^8–10^. A better understanding of the roles and responses of soil biota in various cropping systems is needed to support sustainable agricultural practices^11^.

Nematodes have diverse roles in soils as fungal-feeders, bacterial-feeders, omnivore-predators or plant-parasites, making them valuable bioindicators for assessment of soil health^12–14^. Cropping systems have been shown to have variable effects on soil nematode communities^4,15^, and these changes can provide insight into the functioning of the soil food web^16^. Plant-parasitic nematodes have been widely-studied because of their economic importance, with up to 25% of global crop yield losses attributed to their damage^17^. Other studies have focused on free-living species because of the regulatory influence fungal- and bacterial-feeding nematodes have on decomposition, nitrogen mineralization and microbial communities^18–20^. Predatory nematodes are also of interest because they provide top-down control of plant-parasitic nematodes^21,22^.

Traditionally, nematode communities are studied by extracting the organisms from soils, examining their morphology under light microscopy and using key morphological features to make taxonomic identifications. Morphology-based analysis of nematode diversity and community structure is time consuming, requires extensive knowledge of nematode taxonomy and, for expediency, is often limited to identification at higher taxonomic ranks, such as family or genus^23^. In soil nematode communities, which are species-rich and are thought to locally (e.g., within a field site) contain as many as 100–200 species, a relatively small number of species typically dominate the entire community, with many rare species also present. Identification of these rare species is particularly challenging because it may be difficult to find enough representatives to study (ideally, adult females and males). An alternative approach that mitigates many of these challenges is the use of DNA-based metabarcoding, which has assumed a critical role in biodiversity studies, particularly of microscopic organisms^24–28^, yet has not been applied to the study of nematode communities within agroecosystems. Metabarcoding of nematode communities has the potential to provide increased taxonomic resolution and capture rare taxa that may be missed or misidentified through morphological analysis, thereby providing a more complete picture of nematode diversity and response to land-use changes^29^.

The U.S. Department of Agriculture’s Farming Systems Project (FSP) in Beltsville, Maryland, part of the USDA Long-Term Agroecosystem Research (LTAR) network, is comparing the impact of farming management approaches on productivity, sustainability and economic stability for farmers^30^. The objective of our study was to compare nematode abundance, diversity and community structure in soils at the FSP from plots that represent typical grain production systems in the mid-Atlantic region of the U.S. Using both DNA metabarcoding and morphological approaches, we compared nematode communities in soils from field plots that were in corn: soybean: wheat rotations and were managed conventionally with tillage (CT), conventionally without tillage (NT) and following organic standards with tillage (ORG). Fertility was supplied with mineral fertilizers in CT and NT and with poultry litter and a legume cover crop in ORG. We hypothesized that more diverse organic inputs to the ORG system would promote a more abundant and diverse free-living nematode community, and that the NT system would also show higher diversity and a more structured trophic system than CT due to decreased disturbance of the soils.

## Results

### Description of data from metabarcoding and morphological analyses

**Figure 1:**
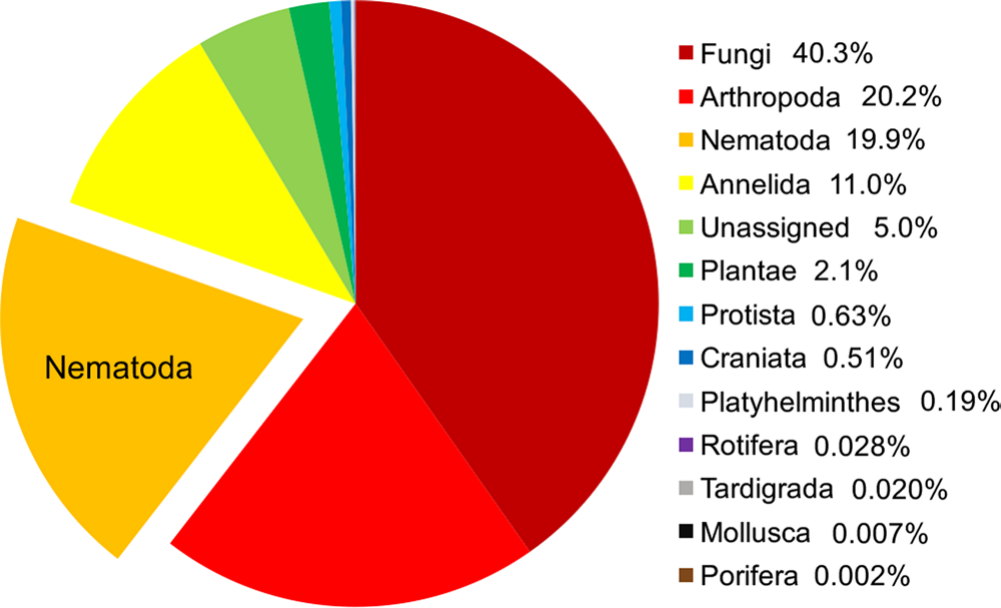
Relative abundance of eukaryotic OTUs obtained via 18S rDNA metabarcoding. All 48 samples from the field experiment are pooled.

**Figure 2:**
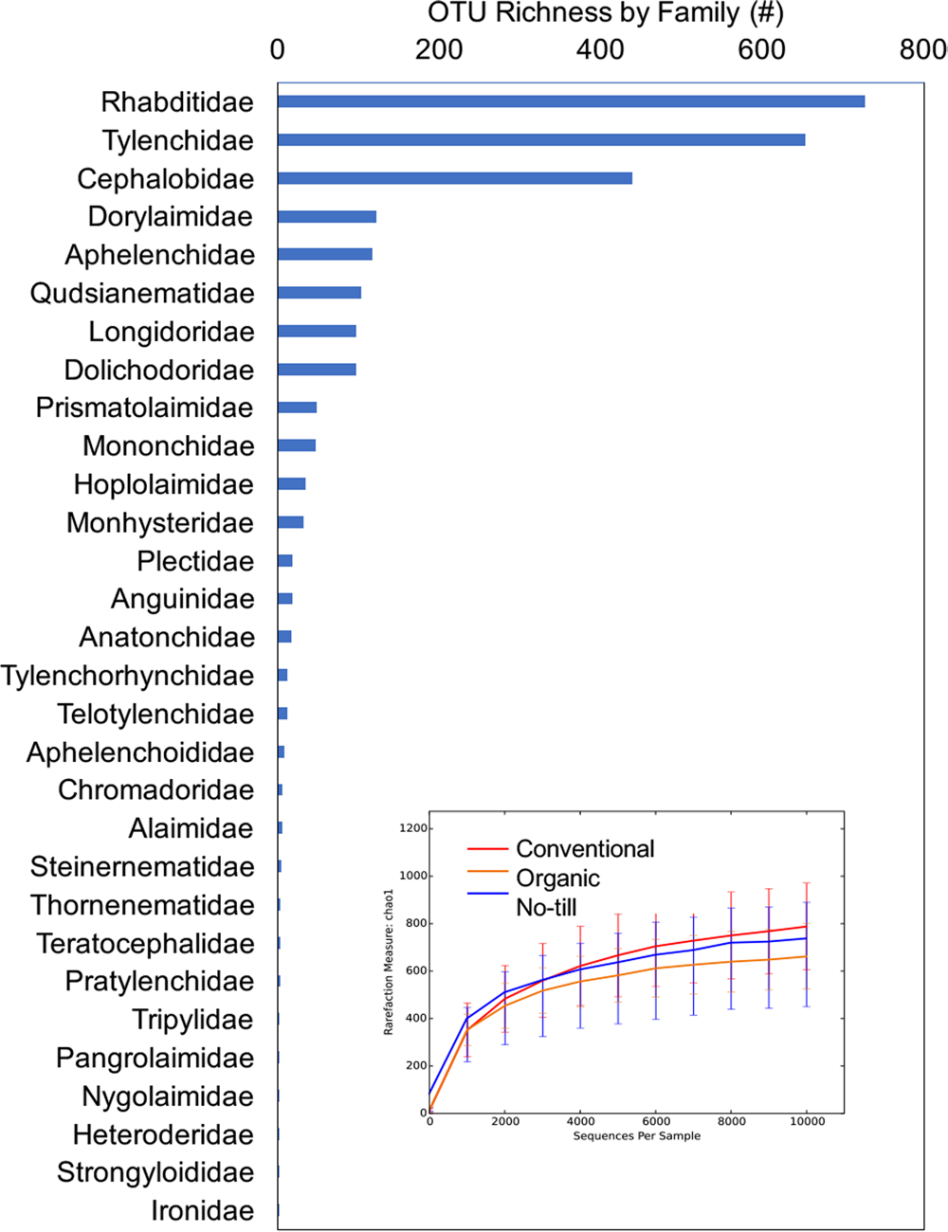
Nematode families ranked by the number of unique metabarcoding operational taxonomic units (OTUs) found across all samples (99% identity threshold, n = 48 samples). Inset: Rarefaction curves for nematode 18S rDNA OTUs obtained for each cropping system. Four samples for which fewer than 10,000 sequencing reads were obtained were omitted from the rarefaction analysis.

Metabarcoding analysis recovered operational taxonomic units (OTUs) from twelve eukaryotic phyla (Fig. 1), with 19.9% assigned to the Nematoda. At a 99% similarity threshold, 3034 unique nematode OTUs were found, encompassing 30 nematode families (Fig. 2). More than half (1628) of the nematode OTUs were identified to genus level or below. Rarefaction curves indicate that much of the potential nematode OTU diversity that could be detected with our primer set was captured (Fig. 2).

**Figure 3:**
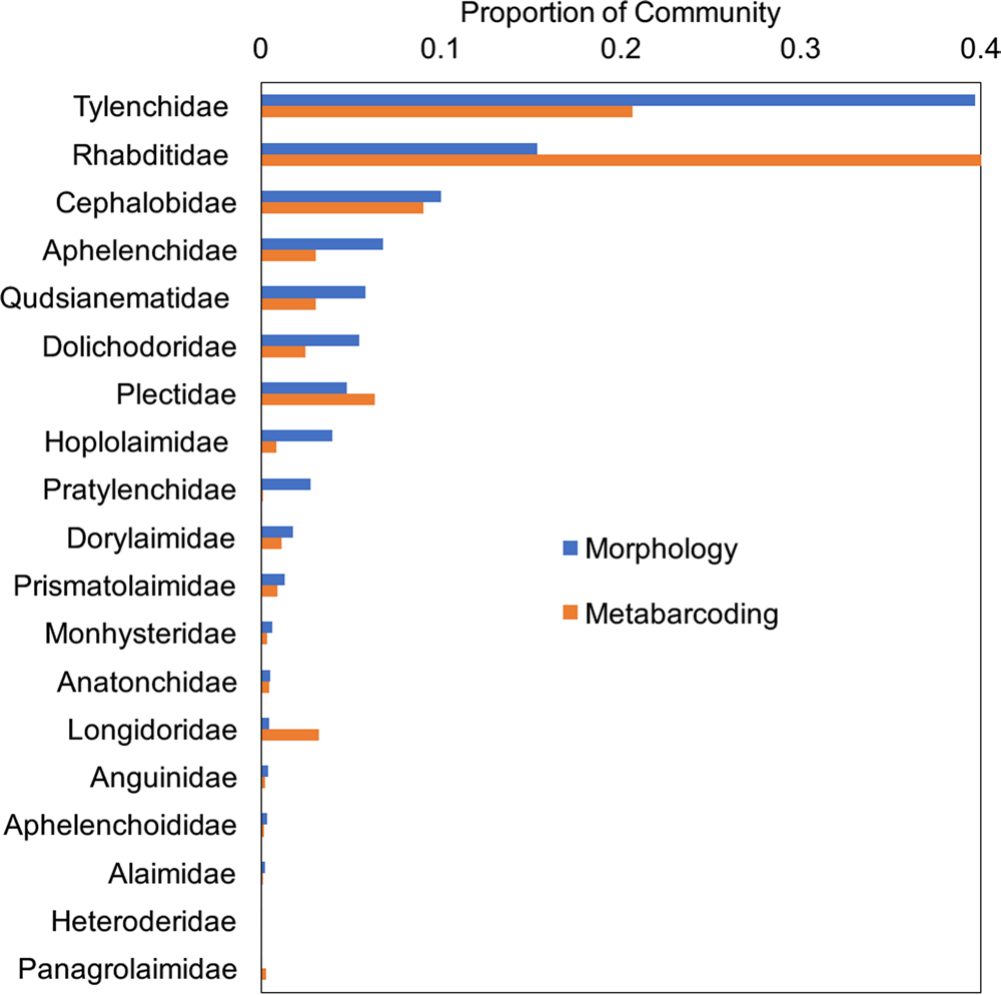
Comparison of representation of families in the nematode community between the morphological and metabarcoding analyses. Values are the means (n = 48) across all treatments and replicates. Only families recovered by both analyses are shown.

Morphological analysis distinguished twenty-one nematode families (Supplementary Fig. S1). Of these families, Paratylenchidae and Criconematidae were not found via metabarcoding analysis. These were each represented by fewer than five individuals among the samples. Several families that were found by metabarcoding but not identified by morphology were nearly all rare (i.e., their OTUs were absent in a majority of samples and when present, represented less than 0.5% of OTUs; Chromadoridae, Ironidae, Steinernematidae, Strongyloididae, Telotylenchidae, Teratocephalidae, Thornenematidae, Trypylidae, Tylenchorhynchidae). The families that were well-represented in the metabarcoding analysis but not identified by morphology were the Mononchidae and Nygolaimidae. If these nematodes were present in the samples studied microscopically, they may have been assigned to closely-related families. For families that were found by both analyses, there were varying degrees of congruence between their proportional abundances (Fig. 3, Supplementary Fig. S1). On average, the Tylenchidae composed 39.7% of the nematode communities by morphological analysis and 20.1% by metabarcoding (Fig. 3). The Rhabditidae composed 15.3% by morphological analysis and 42.9% according to metabarcoding (Fig. 3). The differences between the two approaches were not as striking for other taxa (Fig. 3). However, families that seem to be overrepresented via metabarcoding include the Plectidae (4.8% of the community for morphology vs. 6.3% for metabarcoding) and Longidoridae (0.46% vs. 3.2%), while many of the remaining families are slightly underrepresented (Fig. 3).

### Soil nematode abundance and trophic groups among cropping systems

**Figure 4:**
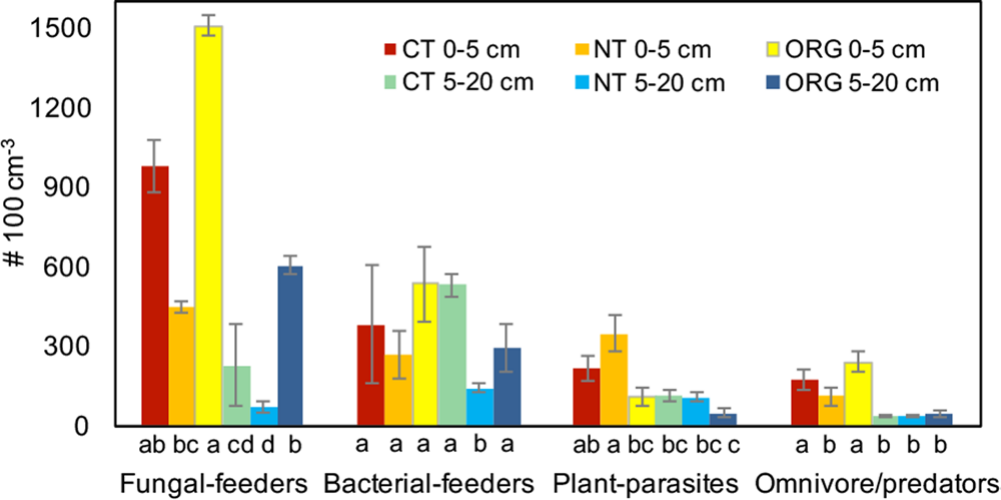
Density of nematodes from four trophic groups among the three cropping systems, by depth (CT = conventional, tilled, NT = no-till and ORG = organic, tilled), according to morphological analysis. Bars represent mean values ( ± s.e.m., n = 8) for each cropping system by depth combination. Within a trophic group, bars that share the same lower-case letters are not statistically different from each other (ANOVA, significant system, depth, system, and/or system×depth effects, *P* < 0.01; Tukey-Kramer test).

Soils from the three cropping systems hosted significantly different nematode communities, with both the morphological and metabarcoding approaches producing similar results (Supplementary Fig. 1). The metabarcode data tended to be more variable across field replicates, obscuring statistically significant treatment effects that were found using the morphology-based approach, although data trends were similar. The greatest contrast in nematode communities was between the ORG and NT systems. Based on microscopic counts, NT had significantly fewer nematodes than ORG at both depths, while CT was intermediate (Table 1). The density of nematodes from different trophic groups varied significantly among cropping systems (Fig. 4). Fungal-feeders were more abundant in ORG than NT in the 0–5 cm layer and more abundant than in CT and NT in the 5–20 cm layer (Fig. 4). At 5–20 cm, bacterial-feeder abundance was lowest in NT, but otherwise similar among the cropping systems (Fig. 4). NT contained more plant-parasites and fewer fungal-feeders than ORG at 0–5 cm (Fig. 4). At 0–5 cm, NT had fewer omnivore-predators than CT or ORG (Fig. 4).

**Table 1:** Nematode density and family-level diversity comparisons among cropping systems*.

	0–5 cm			5–20 cm			ANOVA results
	CT	NT	ORG	CT	NT	ORG	
All nematodes (# 100 cm^−3^, Morphology)	1751.9 ± 368.2 cd	1165.9 ± 148.9bc	2394.1 ± 182.3d	906.9 ± 171.8b	359.7 ± 34.1a	994.0 ± 134.7bc	System***, Weed N. S., Depth***, Interactions N. S.
Tylenchidae (# 100 cm^−3^, Morphology)	801.4 ± 185.4c	497.3 ± 90.6bc	1499.1 ± 145.2d	144.5 ± 32.7ab	50.5 ± 15.3a	541.1 ± 82.2c	System***, Weed N. S., Depth***, Interactions N. S.
Rhabditidae (# 100 cm^−3^, Morphology)	124.5 ± 35.7	59.1 ± 12.4	128.1 ± 24.3	427.1 ± 167.5	59.0 ± 10.58	194.6 ± 25.8	System N. S., Weed N. S., Depth*, System×Depth*
Cephalobidae (# 100 cm^−3^, Morphology)	204.5 ± 53.6	150.2 ± 13.3	331.9 ± 27.9	53.9 ± 9.1	25.6 ± 6.8	54.4 ± 9.5	System*, Weed N. S., Depth***, Interactions N. S.
Bongers MI (Morphology)	1.9 ± 0.07bc	1.6 ± 0.07ab	2.1 ± 0.05c	1.4 ± 0.05a	1.5 ± 0.1a	1.8 ± 0.05bc	System**, Weed N. S., Depth***, Interactions N. S.
Richness (# of families, Morphology)	11.8 ± 0.7	14.0 ± 0.2	10.1 ± 0.5	11.6 ± 0.6	12.0 ± 0.6	10.5 ± 0.5	System N. S., Weed N. S., Depth N. S., Interactions N. S.
Shannon Index (Morphology)	1.6 ± 0.1ab	1.8 ± 0.1bc	1.3 ± 0.05a	1.8 ± 0.1b	2.1 ± 0.5c	1.5 ± 0.1a	System**, Weed N. S., Depth***, Interactions N. S.
Simpson’s Index (Morphology)	0.7 ± 0.02ab	0.8 ± 0.03bc	0.6 ± 0.02a	0.8 ± 0.04bc	0.9 ± 0.01c	0.7 ± 0.03ab	System**, Weed N. S., Depth***, Interactions N. S.
Richness (# of families, Metabarcoding)	18.9 ± 0.9	19.9 ± 0.9	16.9 ± 1.0	17.9 ± 1.2	17.0 ± 0.8	18.0 ± 1.1	System N. S., Weed N. S., Depth N. S., Interactions N. S.
Shannon Index (Metabarcoding)	1.7 ± 0.11	1.94 ± 0.064	1.51 ± 0.09	1.2 ± 0.14	1.6 ± 0.10	1.3 ± 0.11	System N. S., Weed N. S., Depth***, Interactions N. S.
Simpson’s Index (Metabarcoding)	0.7 ± 0.05bc	0.8 ± 0.02c	0.6 ± 0.04abc	0.5 ± 0.06a	0.7 ± 0.05bc	0.6 ± 0.06ab	System N. S., Weed N. S., Depth**, Interactions N. S.

*CT = conventional, tilled, NT = no-till and ORG = organic, tilled. Values are means (±s.e.m., n = 8). Within a row, values with different letters are significantly different (Tukey-Kramer test, *P* < 0.05). Comparisons are only shown for analyses with significant differences among the cropping systems at the *P* < 0.01 significance level. Split-Split plot ANOVA results: ***P < 0.001, **P < 0.01, *P < 0.05, N. S. = not significant.

Three nematode families numerically dominated the communities across the field site: Tylenchidae, Rhabditidae and Cephalobidae. Microscopic counts show that fungal-feeding Tylenchidae density was higher in ORG at both depths as compared to CT and NT (Table 1). Rhabditidae and Cephalobidae density did not vary significantly among the cropping systems (Table 1). Plant-parasitic nematodes represented a relatively small proportion of the communities across the study site (Supplementary Fig. S1). However, the specific plant-parasitic taxa whose density was higher in NT as compared to CT and ORG in the 0–5 cm layer include the Hoplolaimidae (*Helicotylenchus* spp.) and Pratylenchidae (*Pratylenchus* spp.) (data not shown). The Bongers Maturity Index was significantly different among the cropping systems, with the highest values found in ORG at both depths (Table 1).

### Nematode diversity among cropping systems

Family-level taxonomic richness of nematode communities (i.e., the number of families present) was not affected by cropping system or depth (Table 1). However, the Shannon and Simpson’s diversity indexes, which consider both evenness and richness, showed that NT had higher values than ORG at both depths (Table 1). Using the results from metabarcoding to examine differences in nematode diversity at a finer taxonomic scale, OTU diversity was compared among cropping systems for the three most abundant families (Tylenchidae, Rhabditidae and Cephalobidae) (Table 2). OTU richness did not vary among the cropping systems for any of these families (Table 2). However, the Shannon and Simpson index values for the Tylenchidae were higher in ORG than in NT in the 0–5 cm layer (Table 2). For the Rhabditidae, these values were higher in ORG than in CT and NT in the 0–5 cm layer (Table 2).

**Table 2:** Within-family diversity comparisons among cropping systems (metabarcoding only)*.

	0–5 cm			5–20 cm			ANOVA results
	CT	NT	ORG	CT	NT	ORG	
Tylenchidae richness (# of unique OTUs)	216.5 ± 16.2	171.4 ± 21.2	248.1 ± 20.6	116.3 ± 18.0	95.5 ± 29.3	153.6 ± 14.1	System*, Weed N. S., Depth***, Interactions N. S.
Tylenchidae (Shannon Index)	3.4 ± 0.2bc	2.7 ± 0.2ab	3.6 ± 0.06c	2.8 ± 0.2ab	2.1 ± 0.3a	2.9 ± 0.1abc	System***, Weed N. S., Depth***, Interactions N. S.
Tylenchidae (Simpsons Index)	0.90 ± 0.02b	0.83 ± 0.03a	0.93 ± 0.01b	0.87 ± 0.02ab	0.78 ± 0.06a	0.85 ± 0.01ab	System***, Weed N. S., Depth**, Interactions N. S.
Rhabditidae richness (# of unique OTUs)	149.8 ± 24.2	130.6 ± 16.9	65.5 ± 13.6	239.3 ± 33.0	148.1 ± 20.8	163.6 ± 32.4	System N. S., Weed N. S., Depth***, System × depth*
Rhabditidae (Shannon Index)	1.4 ± 0.1a	1.4 ± 0.1a	2.2 ± 0.2b	0.9 ± 0.08a	1.0 ± 0.1a	1.3 ± 0.1a	System***, Weed N. S., Depth***, System × weed*
Rhabditidae (Simpsons Index)	0.4 ± 0.04a	0.5 ± 0.06a	0.7 ± 0.08b	0.3 ± 0.03a	0.3 ± 0.06a	0.4 ± 0.05a	System***, Weed N. S., Depth***, System × weed**
Cephalobidae richness (# of unique OTUs)	171.1 ± 17.0	107.6 ± 11.2	104.3 ± 10.4	118.5 ± 15.1	104.1 ± 16.2	70.8 ± 18.4	System N. S., Weed N. S., Depth*, Interactions N. S.
Cephalobidae (Shannon Index)	3.3 ± 0.2	2.8 ± 0.02	3.4 ± 0.1	2.7 ± 0.2	2.2 ± 0.3	2.6 ± 0.2	System*, Weed N. S., Depth ***, Interactions N. S.
Cephalobidae (Simpson Index)	0.9 ± 0.02	0.8 ± 0.05	0.9 ± 0.02	0.8 ± 0.04	0.7 ± 0.09	0.8 ± 0.04	System*, Weed N. S., Depth*, Interactions N. S.

*CT = conventional, tilled, NT = no-till and ORG = organic, tilled. Values are means (±s.e.m., n = 8). Within a row, values with different letters are significantly different (Tukey-Kramer test, *P* < 0.05). Comparisons are only shown for analyses with significant differences among the cropping systems at the *P* < 0.01 significance level. Split-Split plot ANOVA results: ***P < 0.001, **P < 0.01, *P < 0.05, N. S. = not significant.

### Redundancy analysis (RDA)

**Figure 5:**
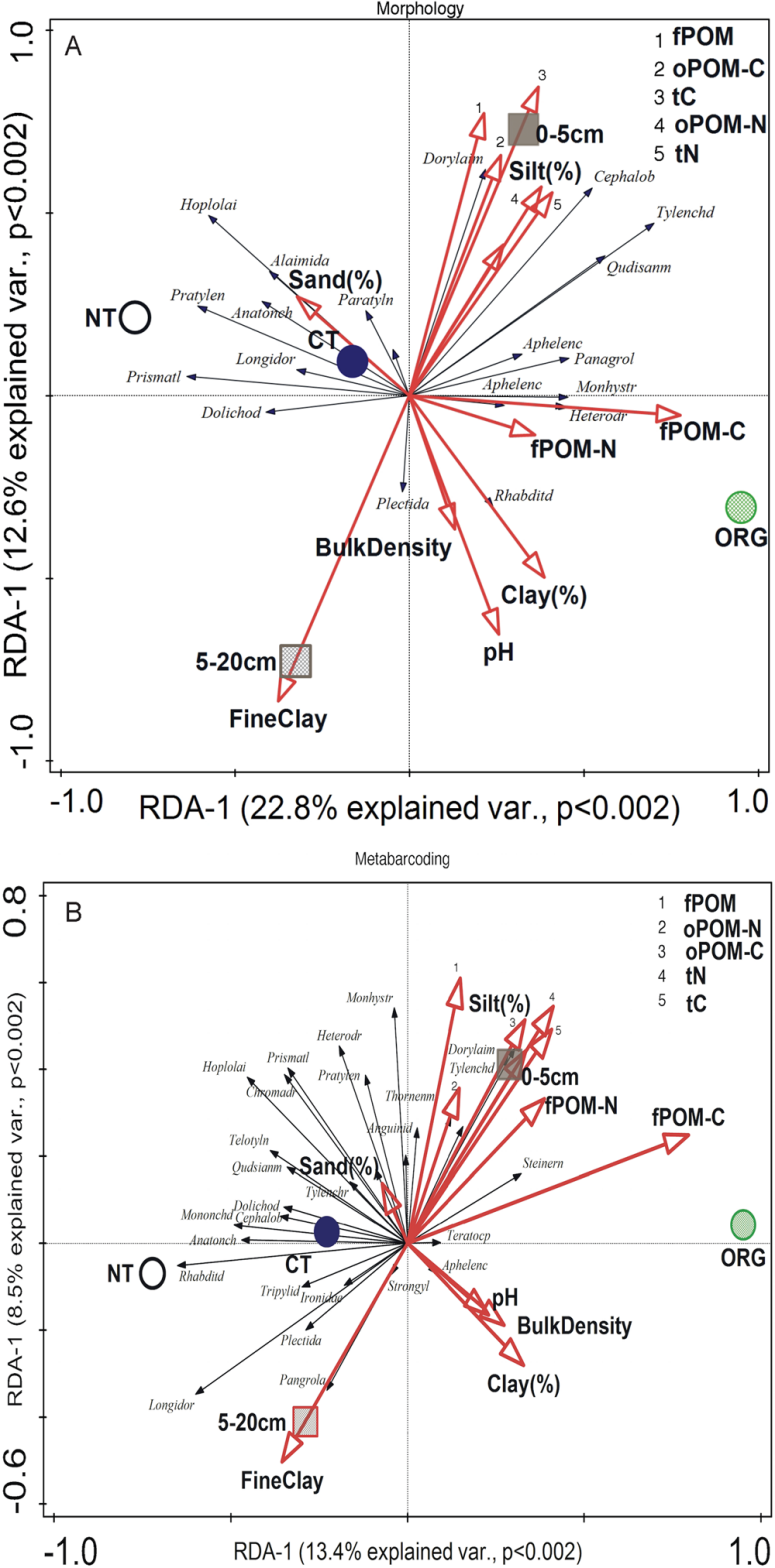
Redundancy analyses of the relationships between nematode community structure, cropping system, and soil environmental properties for (**A**) morphological and (**B**) metabarcoding analyses (CT = conventional, tilled, NT = no-till and ORG = organic, tilled. fPOM = free particulate ORG matter, oPOM = occluded particulate ORG matter, tN = total soil nitrogen, tC = total soil carbon). Axis 1, from top to bottom, is dominated by the effects of depth, with 0–5 cm on the top and 5–20 cm on the bottom. Axis 2, from right to left, is dominated by differences among cropping systems with NT and CT systems to the left and the ORG system positioned to the right.

The results from RDA support the differences in soil nematode community structure among cropping systems described above while also indicating how these changes relate to soil properties (Fig. 5). The morphological and metabarcoding analyses both show that fungal-feeding Tylenchidae and omnivorous Dorylaimidae each associate with increased soil carbon (tC) and nitrogen (tN), fPOM, oPOM-C and -N, and fPOM-C and -N, which were all higher in ORG (Fig. 5). For the morphological analysis, this association extends to the Cephalobidae and Qudsianematidae (Fig. 5). Plant-parasitic nematode taxa (Hoplolaimidae, Pratylenchidae and Dolichodoridae) each associated with the NT system (Fig. 5). The Rhabditidae shift from one side of the ordination to the other between the morphological and metabarcoding analyses, mainly across Axis 2, which reflects differences between the cropping systems (Fig. 5). The large disparity between the approaches with respect to relative representation by this family influences how the group is weighted, impacting its statistical response in the RDA.

## Discussion

Concerns regarding the negative impacts of conventional farming practices on soil biodiversity and function have led to interest in sustainable agriculture and the investigation of how cropping systems affect soil ecosystems. Long-term experiments like the FSP are valuable resources for studying farming practices that sustain the integrity of soil ecosystems and support soil biodiversity. Although confined to a single sampling date, the timing of our study near the end of the wheat growing season allowed us generate a snapshot of the nematode community at a given point in time in three similar crop rotations under very different management practices. Through a combination of morphological and metabarcoding approaches, we determined that cropping systems select for distinct nematode taxa, reflecting the unique ecologies of each system.

The primary goal of reducing tillage is to diminish soil erosion, labor and energy use. A benefit of no-till is an increase in surface soil quality, including greater soil carbon than in tilled systems^31^. Prior work at the FSP field site measured higher soil carbon and aggregate stability in the NT versus the CT system at the 0–5 cm depth^32^. These improvements in soil quality should positively affect the decomposer food web and the density of free-living nematodes. Specifically, no-till practices are believed to enhance the fungal decomposition pathway^33^ and in turn, the density of fungal-feeding nematodes. The NT system also received more herbicide applications than CT, which could affect nematode community structure. Compared to the CT, the NT system had lower abundance of some trophic groups of free-living nematodes (omnivore-predators and bacterial-feeders) but higher abundance of some plant-parasitic taxa, according to morphological analysis. Fungal-feeders did not differ between these two systems. Following at least 29 years of no-till at our field site, only certain plant-parasites show higher abundance in NT than in CT. Interestingly, family-level and OTU-level nematode richness did not differ in NT as compared to CT. Although abundance was lower for some free-living nematodes in NT, the overall diversity of the communities was generally unaffected due to the increased representation of plant-parasitic taxa in these soils.

Changes in the NT cropping system that reduce the abundance of free-living nematode species are of concern, as these nematodes include predatory taxa that help to control plant-parasitic species and microbivorous taxa that are important grazers of the decomposer organisms that generate soluble nutrients for plant uptake^19^. Studies of nitrogen release in the FSP have not shown any differences between the NT and CT systems, however^34^. The effects of no-till on soil nematode communities have been studied by others and the results are somewhat variable^4,15,35–38^, which is likely to be a reflection on the variation among studies in soil types, sampling dates, crop rotations, agricultural inputs and duration of no-till. However, many studies report positive effects of no-till on the diversity and abundance of microbivorous nematodes^36,37^ that we did not find in our study. Most published studies of nematode communities and tillage involve no-till for only five years or less, in contrast to the 29 years of the FSP. It may be that initial gains in soil resources for the decomposer food web from eliminating tillage attenuate over time as resources become increasingly concentrated at the surface layer of the soil. More long-term studies of the impact of this important management strategy on soil nematode communities are needed.

Organic practices focus on enhancing soil organic matter, fertility and the soil food web through use of organic fertilizers and crop rotation with cover crops. Synthetic pesticides and many other chemical inputs that can affect nematodes are excluded from organic practices^6^. In our experiment, the ORG systems were sown with a hairy vetch cover crop and received poultry litter inputs that the NT and CT systems did not receive. Previous studies have shown positive effects of organic farming practices on soil food webs^3,39,40^. We found that the density of some nematode groups, particularly fungal-feeding Tylenchidae, was higher in ORG as compared to the CT system. The poultry litter applied in the ORG system contained partially-decomposed wood chips, which should promote the fungal decomposition pathway and a subsequent increase in fungal-feeding nematodes. Changes in the abundance of Tylenchidae and Cephalobidae were also linked to higher soil carbon, nitrogen and particulate organic matter in ORG, as indicated by RDA analysis.

Ferris and Tuomiso proposed that higher nematode species diversity could enhance ecosystem services and improve soil health^14^. There were no differences between the ORG and CT systems with respect to nematode diversity at the family level (i.e., the richness and evenness of representation of the families). However, according to within-family analyses using OTU data from metabarcoding, the Rhabditidae Shannon and Simpson indexes were higher in ORG soils when compared to CT (0–5 cm). Density and OTU richness of Rhabditidae did not vary. These bacterial-feeding nematodes have short generation times and are “enrichment opportunists”, responding to pulses of organic matter^41^, such as the poultry litter applied in the ORG system. Organic management at our field site appears to have promoted the abundance of a subset of Rhabditidae taxa, increasing the evenness of this nematode family.

Neher found that the soil nematode community structure of organic and conventionally managed cropping systems in North Carolina, USA were very similar to each other after eight years and suggested tillage has a far more significant, and negative, effect on nematode communities than organic vs. conventional management^42^. In our study, the nematode communities in the ORG system were more similar to those in CT than to those in NT, but both were characterized by positive attributes that the NT nematode communities lacked, including higher abundance of bacterial-feeding nematodes at 5–20 cm. The ORG soils had higher Bongers MI values than CT and NT at 5–20 cm, indicating that those soils contained nematode communities associated with less disturbance, with more k-selected taxa. These results do not support our hypothesis that the NT system would have a more structured trophic system (e.g. higher Bongers MI) than the tilled systems due to decreased soil disturbance. The NT system, perhaps due to greater herbicide applications as well as changes in resource distribution, has negatively impacted soil nematode communities at the FSP. ORG plots without tillage were not included in our study, but it is possible that the negative effects on nematode communities that we found in the NT system could be offset to some extent by addition of cover crops and organic amendments.

Our results allow us to evaluate the utility of a metabarcoding approach for the study of how soil nematode communities are affected by management strategies in agroecosystems. We found a degree of congruence between the morphological and metabarcoding approaches as well as instances where the two methods diverge from one another. Inherent to both approaches is the introduction of several sources of error that may mask or introduce variation, affecting diversity estimates and community analyses. With respect to metabarcoding, the choice of a specific gene for analysis is critical^26,29,43^. We selected a validated primer set for soil nematode community analysis that amplifies a fragment of the 18S rDNA gene with strong representation in reference databases (within the nematodes, representation of some taxa remains inadequate, however). 18S fragments that resolve nematode diversity at the level of family or genus may not resolve species-level differences^44,45^. This is because of evolutionary constraints on the gene as well as the lack of information about within-species 18S gene copy number and associated polymorphisms^46,47^. Bik *et al*. used whole-genome shotgun sequencing to show that a single nematode genome may have several hundred repeat copies of the 18S gene, with numerous intragenomic polymorphic positions^48^. Therefore, while use of 18S rDNA fragments amplified by the NF1/18Sr2b primer set may underestimate diversity due to an inability to resolve species differences, it is simultaneously likely to inflate diversity if polymorphisms are identified as originating from separate species^46,48^. Furthermore, use of a double-PCR approach to ensure maximum amplification for metabarcoding could compound these issues through increased introduction of PCR errors or biased amplification of a subset of 18S amplicons. Our bioinformatics workflow removed chimeric sequence artifacts (reads composed of sequence from two or more templates) and rejected OTUs with fewer than ten occurrences, ideally reducing inclusion of erroneous sequences. Ultimately, the high number of unique OTUs that we identified within a single field site (n = 3034; 99% identity threshold) is not likely to be an accurate representation of the number of species present. Overall, our data agree with a study by Darby *et al*. showing that some key soil nematode families are under- (Tylenchidae) or overrepresented (Rhabditidae) in 18S metabarcode libraries^47^. Body size may be a contributing factor explaining why Tylenchidae (small, slender nematodes) are under-represented and the Rhabditidae (larger, more full-bodied) are over-represented by the metabarcoding analysis. Darby *et al*. used estimates of relative 18S gene copy number to correct their metabarcoding data so that it was more consistent with their morphological analyses^47^.

Nematode community analyses using morphological or metabarcoding approaches are also affected by the relative robustness of the extraction method for separation of nematodes or nematode DNA from soils. The primers we selected do not exclusively amplify nematode sequences. Therefore, we used sugar flotation and centrifugation to separate the nematodes from the soil prior to DNA extraction, to limit the inclusion of non-nematode DNA as much as possible. Several researchers conducting sequencing-based studies of eukaryotes, including nematodes, directly extracted environmental DNA from soils and sediments^27,29,49,50^. Nematodes seem to be underrepresented among the metazoan sequences obtained in these studies (e.g., 2.5%^29^), perhaps due to their small size. Despite the physical separation of nematodes from soil prior to isolation of DNA nematodes only represented 19.9% of sequences obtained in our study. Fortunately, non-nematode amplicons do not seem to be impeding our ability to capture the majority of nematode diversity present, as indicated by our rarefaction curves. Non-nematode sequences can mostly be attributed to fungal hyphae and spores and other soil invertebrates that could have been ingested by and/or co-extracted with the nematodes and to extraorganismal DNA that might be washed from the soil. This could explain the presence of mammalian sequences, such as the opossum or rat sequences we obtained. It has been shown by others, and our results support, that a great deal of free-DNA, termed relic DNA, is preserved in soils, long after the whole organism is gone^51,52^. This may be true for nematodes as well as other taxa and could contribute to overestimating nematode diversity in soils. There were a number of nematode families found by the metabarcoding analysis that were not observed morphologically. Most of these were fairly rare, and it remains unclear whether these were actually present in the soil and were not observed, if they were misidentified in the morphological analyses, and/or if they were present as relic DNA only. This is a confounding issue for many studies that characterize biological communities based on eDNA. In the future, as genome-scale data becomes available from representative taxa across the Nematoda, integrating transcriptome analysis into metabarcoding studies could help to confirm the presence of living organisms^53^.

Metabarcoding has potential to complement or even replace time-consuming morphological-based analyses, but more research is needed to resolve the issues identified above. Revisions to the workflow with the intention of minimizing errors are necessary before this technique can be readily implemented into studies of soil ecology. Furthermore, there is a need for expansion and curation of nematode 18S rDNA databases, in order to facilitate accurate identification. Metabarcoding should remain a method that is used alongside morphology-based analyses until it has been improved such that it can be considered a suitable replacement.

Achieving increases in food crop productivity while simultaneously reducing intensity of crop supplements depends in part on better understanding and management of organisms providing ecological services in agricultural environments. Despite the importance of nematodes in nutrient cycling across trophic levels and as major factors in crop loss, their ecology in agricultural soils remains poorly understood. To overcome the challenges of working with microscopic, morphologically-reduced organisms, we employed multiple strategies to survey nematode communities. We find consistent support for distinct communities present in agricultural soils under differing management strategies using both molecular and morphological identification methods. Long-term study plots such as the FSP are vital to understanding the factors driving such differences owing to accumulated background data and extensive characterization of physical and chemical attributes. Continued building of foundational knowledge of nematode communities requires sampling across broader geographic scales at multiple time points throughout the season, as well as improvements in the methods available for nematode identification.

## Methods

### Study site and soil sampling

The FSP plots were established in 1996 in a field that had been in no-till crop production since 1985. The FSP soils are Coastal Plain silty loam Ultisols, consisting primarily of Christiana, Keyport, Matapeake and Mattapex soil map units^34^. The FSP includes three cropping systems, replicated in four blocks, all in a corn: soybean: wheat rotation: 1) CT, a 3-year, conventional chisel-till rotation 2) NT, a 3*-*year, conventional no-till rotation, and 3) ORG, a 3-year, chisel-till and moldboard-plowed organic rotation. The CT and NT systems follow wheat with soybeans whereas the ORG system uses hairy vetch. A cereal rye cover crop was planted after corn in all three systems. The CT and NT systems both received mineral fertilizer and herbicide applications based on University of Maryland recommendations, with the NT system receiving more herbicides than CT. Poultry litter was used as a fertilizer in the ORG system. Field management activities for each system and application rates for inputs can be found in Supplementary Table 1.

Soils for nematode analyses were sampled 9 June 2014 from the wheat phase of the cropping systems. Wheat was at or near maturity at the time of sampling. Within each main plot, samples were collected from two subplots that have been managed with and without weeds since 2009 to investigate the impact of weeds on crop yield and soil properties^54^. From each 15 × 30 ft subplot, six cores were collected from random points at least 2 ft from the plot edges, to a 20-cm depth using a 2.5-cm diameter soil corer. These cores were divided into 0–5 cm and 5–20 cm depth sections, and each section was pooled across the six cores to create a composite soil sample. In total, 48 samples were collected (3 cropping systems × 2 depths×2 weed treatments×4 blocks/replicates).

### Soil analyses

Additional soil cores were collected separately from the same subplots for measurement of soil properties. Bulk density was calculated by dividing the oven-dry corrected mass of composited 0–5 cm and 5–20 cm soil depth fractions from each plot by the calculated volume of the soil cores. Soil texture was determined using a simplified particle size analysis method from the Cornell Soil Health Assessment^55^. Suspensions of 5 g of soil in 10 ml of deionized H_2_O were equilibrated for 30 min, and pH was measured with an Accumet AB15 soil pH meter with a glass electrode (Fisher Scientific, Waltham, MA). Total C and N from air-dry soil samples were measured using dry combustion on a Costech ECS 4010 CHN elemental analyzer (Valencia, CA). NO_3_^−^ and NH_4_^+^ were extracted from 5 g of field-moist soil simultaneously using 25 ml of 1 M KCl. Soil suspensions were shaken for 1 h on a reciprocating shaker and filtered through Whatman #2 filters into scintillation vials. Extractable NO_3_^−^ and NH_4_^+^ were quantified using a Lachat autoanalyzer (Lachat, Loveland, CO).

Free particulate ORG matter (fPOM) and occluded particulate ORG matter (oPOM) were isolated from soils collected from a previous sampling of the same plots in 2012^56^. Soils were sampled at increments of 0–5, 5–10 and 10–20 cm, and information from the 5–10 and 10–20 cm increments were combined across samples using weighted means. For each increment, 40 g of sieved soil (2 cm) were weighed into a 150 ml Nalgene (Nalge Nunc Int’l Corp., Rochester, NY) bottle, 75 ml of high density sodium polytungstate (NaPT) (1.7 g m^−3^) was added and the sample was shaken for 1 h at 100 rpm. The slurry was transferred to a 250-ml beaker and allowed to settle for 16 h, resulting in two distinct phases of approximately equal volume, a light phase that contained fPOM floated to the surface of the solution (i.e., lighter than 1. 7 g m^−3^) and a heavy phase that contained the soil mineral matrix and any associated oPOM. The oPOM and fPOM were separated through a combination of further aspiration and dispersion, dried at 60 °C for 24 h, and weighed. POM-carbon and POM-nitrogen were determined for each fraction on a Costech ECS 4010 CHN elemental analyzer.

### Nematode extraction

Nematodes were extracted from four, replicate, 40-g subsamples from each composite sample using sugar flotation and centrifugation technique^57^. Three of these extractions were combined for metabarcoding analysis, and the fourth was preserved in 5% formalin for morphological analysis.

### DNA extraction and amplification

The three combined nematode extractions were concentrated to 0.5 ml in water prior to DNA extraction, which was done using the MO BIO UltraClean^®^ Tissue & Cells DNA Isolation kit (MO BIO Laboratories Inc., Carlsbad, CA) following the manufacturer’s instructions. PCR amplification of nematode DNA was performed over two steps. First, an ≈ 300 bp fragment of the 18S rDNA gene was amplified using 3 μl extracted DNA template in 25 μl reactions containing 12.5 μl GoTaq® Green Master Mix (Promega, Madison, WI), 8 μl sterile water, 0.75 μl each of 10 μM primers NF1 (*TCGTCGGCAGCGTCAGATGTGTATAAGAGACAG-*GGTGGTGCATGGCCGTTCTTAGTT) and 18Sr2b (*GTCTCGTGGGCTCGGAGATGTGTATAAGAGACAG-*TACAAAGGGCAGGGACGTAAT)^58,59^. The primers’ Illumina sequencing adapters are given in italics above. The PCR protocol consisted of denaturation at 94 °C for 10 min, followed by 35 cycles of denaturation at 94 °C for 60 s, annealing at 58 °C for 30 s, and extension at 72 °C for 1 min with a final elongation at 72 °C for 10 min. A positive control consisting of DNA extracted from a laboratory culture of the nematode *Acrobeloides uberrinus* was also amplified, and negative controls consisting of purified, nuclease-free water were included for each PCR reaction. PCR products were verified on 0.8% agarose gels after staining with ethidium bromide.

To increase the amplicon pool concentration for sequencing, the product from the first 18S rDNA amplification was re-amplified in a second PCR using 7 μl DNA template from the first PCR step (diluted 1:10000 in TE buffer) in 35 μl reactions containing 17.5 μl Phusion Flash High-Fidelity PCR Master Mix (Thermo Fisher Scientific, Waltham, MA), 8.75 μl sterile water, 0.875 μl each of 10 μM primers NF1 and 18Sr2b. The PCR protocol consisted of denaturation at 98 °C for 10 s, followed by 23 cycles of denaturation at 98 °C for 1 s, annealing at 67.6 °C for 5 s, and extension at 72 °C for 8 s, with a final elongation at 72 °C for 1 min. The PCR products were verified on a 0.8% agarose gel.

### High throughput sequencing

PCR products from nematode communities were indexed using the Nextera XT DNA Library Preparation Kit (Illumina, Inc., SanDiego, CA). The final DNA concentration for the samples averaged 65 ng μl^−1^ (range 28.6–83.7). The nematode libraries (n = 48) were combined into a single library with independently indexed 16S amplicons from the same soil samples (n = 48, data not reported). The library was sequenced using 2 × 300 bp paired-end Illumina sequencing (n = 96 indexes) on the MiSeq platform at the Center for Genome Research and Biocomputing (CGRB) at Oregon State University.

### Bioinformatics

Scripts available in the MacQIIME package were used to manage initial processing and analysis of the resulting Illumina amplicons. Bi-directional paired-end reads were joined with the program fastq-join^60^ and then split into separate sequence and quality score files via the scripts “join_paired_ends” and “convert_fastaqual_fastq.py”, respectively. Quality filtering of the joined reads was performed using the default parameters for the “split_libraries.py” QIIME script. Chimeric sequences were identified *de novo* without using a reference database with UCHIME v6.1, using the script “identify_chimeric_seqs.py”. After running “split_libraries.py”, 8,314,925 sequences were retained. Of these, *de novo* chimera checking detected 459,327 potentially chimeric sequences, which were excluded from subsequent analyses, resulting in a total of 7,855,598 sequences used for OTU picking and taxonomy assignment.

OTU picking was performed with the “pick_open_reference_otus.py” script, with the QIIME formatted version of the SILVA 111 database used for the eukaryote-only reference sequences, skipping the alignment and tree building steps. The minimum sequence per OTU threshold was set to ten. OTU similarity was set to 99%, which has been supported through other studies of nematode diversity^26,46,49,61^. Taxonomy was assigned with the SILVA 99% similarity eukaryote-only taxa map file. The portion of OTUs identified as “Nematoda” were then separated with “split_otu_table_by_taxonomy.py”, and estimates of total diversity were graphed for each cropping system with “core_diversity_analyses.py”. Nematode sequences that were not identified to the family level were omitted from analyses of the effect of field treatments on nematode community structure. The workflow was managed using custom Perl scripts employing modules from BioPerl^62^ and the Bio-Community toolkit^63^. The scripts are available for download on Github at the link: https://github.com/rmkepler/FSP_script_repository/tree/master/nematode_18s.

### Nematode enumeration and identification by morphology

Formalin-preserved nematodes were counted using a Zeiss Axiovert 40 CFL inverted microscope (Carl Zeiss MicroImaging, Inc., Thornwood, NY, USA). On average, these 40 g soil samples each yielded 312 individual nematodes (range: 68–1200). Bulk density measurements from the field plots were used to calculate nematode densities (# 100 cm^−3^). An average of 204 nematodes per sample (range = 68–307) were identified to the family or genus level using morphological features under 50–400 × magnification. A more detailed analysis at higher magnification was not performed with the knowledge that these communities would also be studied via metabarcoding.

Nematodes were assigned to trophic groups based on Yeates *et al*., allowing for comparison of the densities and proportional representations of bacterial-feeders, fungal-feeders, omnivore-predators and plant-parasites^64^. There is uncertainty with respect to the feeding behavior of some nematode groups, especially for genera from the root-associated Tylenchidae and Aphelenchoididae, species of which may be fungal-feeding, plant-parasitic or both^64,65^. Species from these groups were classified as fungal-feeders for this study.

Several diversity and ecological indexes were calculated using the results from the morphological and metabarcoding analyses, including richness (i.e., the number of families represented or number of unique OTUs), Shannon^66^ and Simpson’s^67^ diversity indexes, and the Bongers Maturity Index^68^, which is specific to nematode community analysis. The Shannon and Simpson’s diversity indexes each consider the relative abundance of each taxa (“evenness”) as well as richness. For both of these indexes, higher values are considered to represent higher biological diversity. According to Bongers, nematode families that respond rapidly to nutrient inputs due to high fecundity are considered to be colonizers, and taxa that are long-lived and slow to establish, requiring stable conditions, are considered to be persisters^68^. Using these characteristics, Bongers assigned values to free-living, non-parasitic soil nematode families along a c-p scale (1–4, colonizer-persister), which, in conjunction with the relative abundance of the families, are used in the calculation of the Bongers Maturity Index^68^. This index can be used to draw conclusions regarding the soil condition. Higher values are found in more stable environments with low levels of nutrient enrichment, as would be found in an undisturbed, unpolluted natural ecosystem^68^.

### Statistical analyses

Statistical analyses were performed with R version 3.3 (https://www.r-project.org). ANOVA was used to investigate differences in variables among treatment groups (i.e., cropping system, weed removal, and soil depth) using a split-split plot design. The Shapiro-Wilk test was used to assess all dependent variables for normality, which were subsequently log- or square root-transformed as needed. According to ANOVA, there were no differences for the vast majority of variables between the weedy and weed-free sub-treatments, and data from these subplots were combined for figures, tables and *post hoc* comparisons. Only ANOVA results with a level of significance *P* < 0.01 were subjected to *post hoc* analysis (Tukey-Kramer Test), to account for the large number of comparisons. Multivariate analysis was used to describe patterns in the structure of the nematode community in response to experimental and environmental factors. Using data from either the morphological or metabarcoding analyses of nematode community composition, a constrained linear canonical ordination was conducted (i.e., redundancy analysis or RDA) using CANOCO ver. 5.0 (Microcomputer Power, Ithaca, NY, USA). The nematode families were represented as proportional data to facilitate more accurate cross-method comparisons among the ordinations. Experimental and soil environmental factors significantly impacting the structure of the soil nematode community were selected using a forward selection multiple linear regression model (∝ = 0.05). In addition to calculating the total variance explained by each ordination, partitioning of explained variance was conducted with groups of environmental factors to determine the contribution of these factors to the overall structure of the soil nematode community. Factor groups included soil depth (0–5 or 5–20 cm), cropping system (CT, NT, or ORG) and soil attributes (pH, f/oPOM, bulk density, f/oPOM-C & N, total C&N). The datasets generated by current study are available from the corresponding author on reasonable request.

Mention of a trademark of a proprietary product does not constitute a guarantee, warranty or endorsement by the United States Department of Agriculture and does not imply its approval to the exclusion of other suitable products.

## Electronic supplementary material

SupplementaryInformation
