# Supplementary material for: Characterization of soil nematode communities in three cropping systems through morphological and DNA metabarcoding approaches
Source: Sci Rep. 2018 Jan 31;8:2004. doi: 10.1038/s41598-018-20366-5 (PMC5792604; doi:10.1038/s41598-018-20366-5)

Supplementary Material

Characterization of soil nematode communities in three cropping systems through morphological and DNA metabarcoding approaches

Amy M. Treonis, Samantha K. Unangst, Ryan M. Kepler, Jeffrey S. Buyer, Michel A. Cavigelli, Steven B. Mirsky, Jude E. Maul

Supplemental Table 1. Field management activities, 2012 to time of soil sampling in 2014 for the conventional-till (CT), no-till (NT) and organic (ORG) cropping systems at the USDA-ARS Farming Systems Project, Beltsville, Maryland.

System	Year	Crop*	Tillage	Herbicide applications	Fertilizer and poultry litter applications**
CT	2012	Corn-rye	Chisel plow, Disk, Field cultivator, Cultipacker	S-metalochlor (1.42 kg ha^{-1}), Atrazine (0.84 kg ha^{-1}), Callisto (0.76 kg ha^{-1})	Boron (1.1 kg ha^{-1}), UAN (151 kg N ha^{-1}), K_2SO_4 (112 kg K_2O ha^{-1})
	2013	rye-Soybean-Wheat	Spring—Chisel plow, Disk, Cultipacker<br>Fall—Chisel plow, Disk, Field Cultivator, Cultipacker	Spring—Glyphosate (1.12 kg ha^{-1})	Soybean—TSP (56 kg P_2O_5 ha^{-1}), K_2SO_4 (56 kg K_2O ha^{-1})<br>Wheat—NH_4NO_3 (34 kg N ha^{-1}), TSP (56 kg P_2O_5 ha^{-1}), K_2SO_4 (112 kg K_2O ha^{-1})
	2014	Wheat		Thifensulfuron-methyl (18 g ha^{-1}), Tribenuron-methyl (9 g ha^{-1})	UAN (90 kg N ha^{-1}), K_2SO_4 (112 kg K_2O ha^{-1})
NT	2012	Corn-rye		Paraquat (0.90 kg ha^{-1}), Simazine (1.79 kg ha^{-1}), 2,4-D (0.42 kg ha^{-1}), S-metalochlor (1.42 kg ha^{-1}), Atrazine (0.84 kg ha^{-1}), Callisto (0.76 kg ha^{-1})	Boron (1.1 kg ha^{-1}), UAN (151 kg N ha^{-1}), K_2SO_4 (112 kg K2O ha^{-1})
	2013	rye-Soybean-Wheat		Spring—Paraquat (0.71 kg ha^{-1}), Glyphosate (1.12 kg ha^{-1}), First Rate (1.42 kg ha^{-1})<br>Fall—Paraquat (0.71 kg ha^{-1})	Soybean—TSP (56 kg P_2O_5 ha^{-1}), K_2SO_4 (112 kg K_2O ha^{-1})<br>Wheat— NH_4NO_3 (34 kg N ha^{-1}), TSP (56 kg P_2O_5 ha^{-1}), K_2SO_4 (112 kg K_2O ha^{-1})
	2014	Wheat		Thifensulfuron-methyl (18 g ha^{-1}), Tribenuron-methyl (9 g ha^{-1})	UAN (90 kg N ha^{-1}), K_2SO_4 (112 kg K_2O ha^{-1})
ORG	2012	hairy vetch-Corn-rye	Moldboard plow, Disk, Field cultivator, Cultipacker, Rotary hoe, Cultivator		PL (5.04 Mg ha^{-1})
	2013	rye-Soybean-Wheat	Spring—Chisel plow, Cultipacker, Rotary hoe, Cultivator<br>Fall—Chisel plow, Disk, Field Cultivator, Cultipacker		Soybean—K_2SO_4 (56 kg K_2O ha^{-1})<br>Wheat—PL (2.24 Mg ha^{-1}), Gypsum (224 kg ha^{-1})
	2014	Wheat			K_2SO_4 (56 kg K_2O ha^{-1})

*First letter of cash crops capitalized; first letter of cover crops not capitalized

**UAN = urea ammonium nitrate, TSP = triple super phosphate, PL = poultry litter

Supplemental Figure 1. Proportional representation of nematode families organized by treatment and soil depth from the A) morphological and B) metabarcoding community analyses. Bars in shades of red represent fungal-feeders, yellow/orange bars are bacterial-feeders, green bars are plant-parasitic, and blue bars are omnivore-predators. Bars represents the mean values (n = 8) for each cropping system by depth combination.

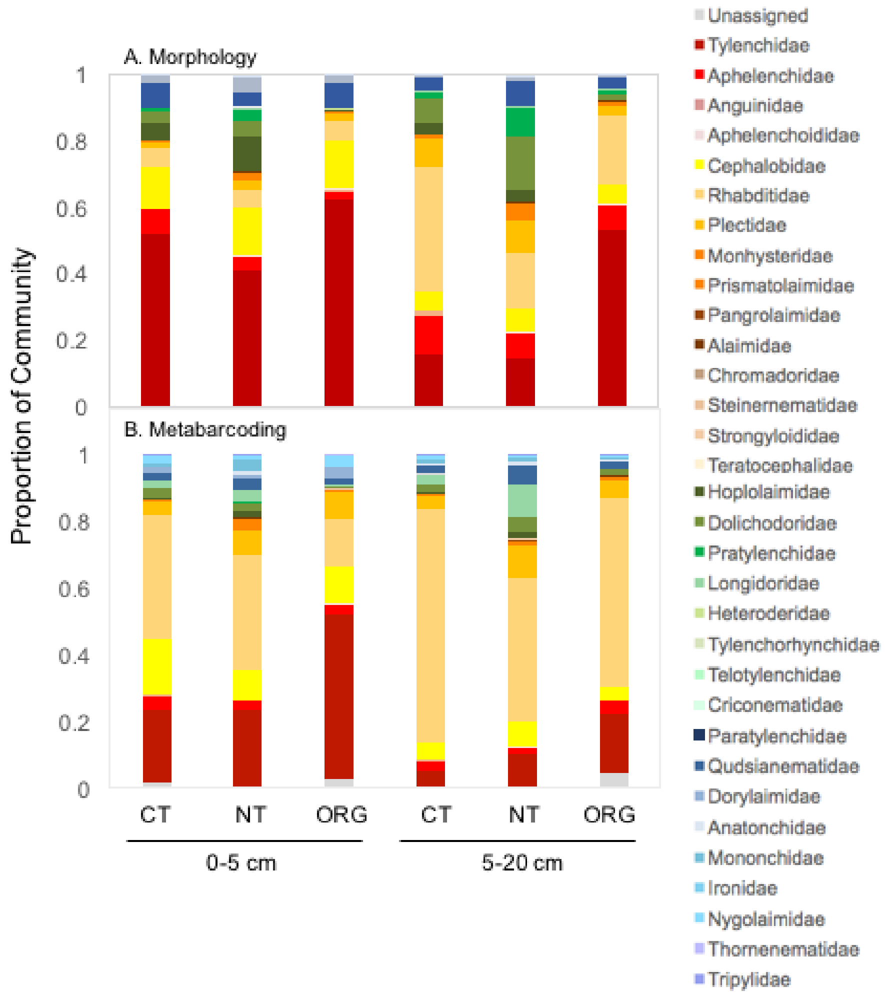

Supplement: Supplementary file 1 — SupplementaryInformation [file 41598_2018_20366_MOESM1_ESM.pdf]
